# Timing of bronchoscopy and application of scoring tools in children with severe pneumonia

**DOI:** 10.1186/s13052-023-01446-3

**Published:** 2023-04-07

**Authors:** Xiangtao Wu, Weihong Lu, Xinquan Sang, Yali Xu, Tuanjie Wang, Xiaowen Zhan, Jie Hao, Ruijuan Ren, Hanshi Zeng, Shujun Li

**Affiliations:** 1 Department of Pediatrics, the First Affiliated Hospital of Xinxiang Medical University, No. 88 of Jiankang Road, Weihui, 453100 Henan province China; 2 Department of Pediatrics, the First Affiliated Hospital of Xinxiang Medical University, Weihui, 453100 China; 3 Department of Pediatrics, Guangdong Provincial People’s Hospital, Guangdong Academy of Medical Sciences, Guangzhou, 510000 China

**Keywords:** Children, Severe pneumonia, Bronchoscopy, Endoscopic scoring, Survival analysis

## Abstract

**Background:**

There is still a lack of effective scoring criteria for assessing the severity of pulmonary infection associated with changes in the endobronchial lining of the bronchus in children. This study aimed to ascertain the timing and value of endoscopic scoring of fibreoptic bronchoscopy (FOB) and bronchoalveolar lavage (BAL) in children with severe pneumonia.

**Method:**

The clinical data of 229 children with severe pneumonia treated with BAL in the Pediatric Intensive Care Unit of the First Affiliated Hospital of Xinxiang Medical University between November 2018 and December 2021 were collected. According to the severity of the disease, patients were divided into an invasive ventilation group and a non-invasive ventilation group, as well as an early BAL group (receiving BAL within 1 day of admission) and a late BAL group (receiving BAL 2 days after admission). A Student’s t-test, Chi-square test, receiver operating characteristic (ROC) curve and survival curve were used to analyse the bronchitis score, aetiology of BAL fluid and survival data.

**Results:**

The scores of endoscopic mucosal oedema, erythema and pallor and the total score in the invasive ventilation group were higher than those in the non-invasive ventilation group (*P* < 0.05), and they were consistent with the Sequential Organ Failure Assessment (SOFA) scores. The secretion colour score was lower in the early BAL group than in the late BAL group (*P* < 0.05). On the bronchitis scores, which were evaluated using a ROC curve, the difference in the mucosal erythema, pallor, oedema and total score of the invasive and non-invasive groups was statistically significant (*P* < 0.05), which was consistent with the area under the ROC of the SOFA scores. Acute Physiology and Chronic Health Assessment II and SOFA scores after FOB were lower than those before treatment (*P* < 0.05). In terms of ICU hospitalisation days and total hospitalisation days, the time of the early FOB patients was shorter than that of the late FOB patients (*P* < 0.05). A total of 22 patients (9.61%) died. The Kaplan–Meier analysis and log-rank test showed that the survival rate of the non-invasive ventilation group was higher than that of the invasive ventilation group (*P* < 0.05).

**Conclusion:**

This study found that FOB combined with BAL is an important method for the diagnosis and treatment of severe pneumonia. Early BAL can reduce hospitalisation and ICU time; however, it cannot improve the survival rate. The endoscopic score has a certain role to play in assessing the severity of pulmonary inflammation, but studies with a large sample are still needed to confirm this.

## Background

Fibreoptic bronchoscopy (FOB) and bronchoalveolar lavage (BAL) help diagnose many pulmonary diseases in children [1, 2] and are often used in paediatric patients with lower respiratory tract lesions and suspected pulmonary infections [3], and even in the assistance of cardiopulmonary surgery [4]. Overall, BAL is considered to provide valuable diagnostic information, and the safety of FOB in paediatric populations and even neonates has been demonstrated [5]. In the paediatric intensive care unit (PICU), FOB is generally used to identify the aetiology of acute respiratory failure in children, such as laryngeal obstruction and foreign bodies [1], and the study of bronchoalveolar lavage fluid (BALF) can assist in early identification of lung disease, pathogens and causes of partial infiltration [5]. Rejection in children who have undergone cardiopulmonary transplantation can be assessed by cellular changes in BALF [6]. In recent years, the use of FOB and BAL in paediatric patients has increased significantly, and the use of FOB and BAL in the PICU has received increasing attention; however, the impact of decisions about their management has not been fully evaluated in previous studies [7] on the diagnosis and treatment of pulmonary diseases. The timing of FOB and BAL use in the treatment of mechanically ventilated patients remains controversial, and the rate of diagnosis in critically ill patients has varied across studies [4, 8]. There is still a lack of effective scoring criteria for assessing the severity of pulmonary infection associated with changes in the endobronchial lining of the bronchus in children.

This study retrospectively analysed the clinical data of 229 paediatric patients with severe pneumonia treated with FOB combined with BAL to investigate the value of endoscopic bronchitis scores and assess the timing of BAL.

## Methods

### Case definition and identification

After applying inclusion and exclusion criteria, this study included 229 patients with severe pneumonia admitted to the PICU of the First Affiliated Hospital of Xinxiang Medical University hospital between November 2018 and December 2021. The inclusion criteria were as follows: the patient met the criteria for severe pneumonia [9]; the indications for the need for BAL (e.g. radiographically confirmed large lung lesions, lung consolidation and atelectasis) were at the discretion of the attending physician; and the patient was being treated in the PICU for the first time. The exclusion criteria were as follows: the patient had a chronic disease (e.g. bronchiectasis or asthma) or malignant tumour; the ICU admission took place after cardiac arrest; respiratory failure had followed solid organ transplantation or surgery; the patient had confirmed or suspected active pulmonary tuberculosis; the patient had immunodeficiency and had been taking immunosuppressive drugs before admission; the patient had an intolerance of BAL; the parents or guardians did not agree; and the patient’s medical data was incomplete. This study was approved by the Ethics Committee of the First Affiliated Hospital of Xinxiang Medical University (No. 2,020,252).

**Fig. 1:**
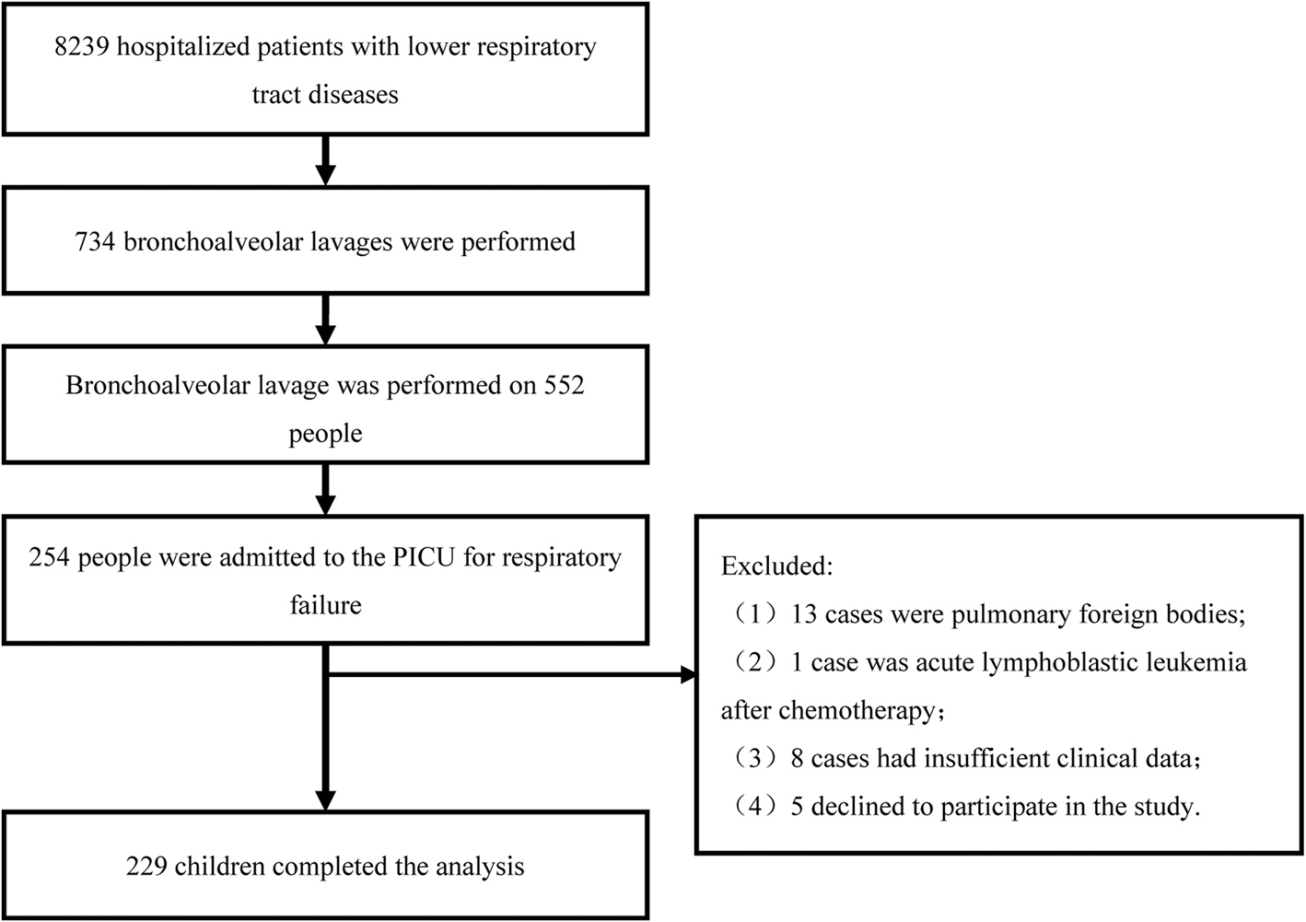
Research flow chart

A total of 552 patients underwent 734 FOB and BAL, and 13 patients had foreign bodies in the lungs. A total of 254 patients were admitted to the ICU due to respiratory failure and pulmonary infection and underwent bronchoscopy and BAL without transbronchial biopsy. One patient with acute lymphoblastic leukaemia after chemotherapy and eight patients with insufficient clinical data were excluded; five patients did not wish to participate in the study. Finally, 229 patients, who underwent a total of 350 bronchoscopies, met the inclusion criteria (Fig. 1).

### Research design

This retrospective observational study was conducted in accordance with the Strengthening the Reporting of Observational Studies in Epidemiology guidelines [10].

The patients were divided into invasive and non-invasive ventilation groups according to disease severity, as well as an early BAL group (received BAL within 1 day of admission) and a late BAL group (received BAL 2 days after admission). The following data were collected from medical records for each patient: demographics (age, sex), time of onset before ICU admission, reason for ICU admission, Acute Physiology and Chronic Health Assessment (APACHE) II score and Sequential Organ Failure Assessment (SOFA) score, length of hospital stay and length of ICU stay. Each patient’s clinical presentation, laboratory test results (BALF microbiological test results, (polymerase chain reaction (PCR) and bacterial culture, 95%; next generation sequencing results, 5%) and chest imaging features were recorded, and the FOB endoscopic score was re-analysed. (Bacteria and fungi were identified by PCR after common culture and selected medium culture. Mycoplasma, virus and other non-bacterial pathogens were directly identified by PCR.)

### Flexible bronchoscopy with BAL

In all patients, bronchoscopy was performed under combined sedation. Based on the authors’ own exploratory experience, a combination of midazolam (0.1–0.3 mg/kg/session, specification 1 ml/5 mg, Jiangsu Nhwa Pharmaceutical Co., Ltd., Xuzhou, China, Guoyao Zhunzi, H19990027) and propofol (specification 20 ml/200 mg, Beijing Fresenius Kabi Pharmaceutical Company Limited, Beijing China, J20130013) was used in all the procedures. In most cases, after one session of combined sedatives, a satisfactory sedative effect was achieved, which lasted until the end of the operation. All the patients underwent FOB and BAL via a tracheal tube in accordance with standard practice in the Guidelines for the Operation of Flexible Bronchoscopy in Children. BAL samples were taken from the lung segment that had the most pathological changes according to a computed tomography (CT) scan. Lidocaine was instilled into the operative port to reduce cough reflex and airway spasm, and intravenous midazolam and propofol were used for moderate sedation. However, atropine was not used to reduce airway secretions. BAL was performed on the most affected areas, as determined by radiology and/or endoscopy. Irrigation consisted of infusing 3–5 portions of sterile saline solution heated to 37 °C at 1 ml/kg/time, followed by immediate aspiration. The fluid was recovered at a negative pressure of 6.65–13.3 kPa (50–100 mmHg). The recovery of BAL liquid was at least 40%. Multiple FOB and BAL procedures were performed during the same hospital stay on a small number of patients.

### Endoscopy score tool

The treating physician performed FOB by using an Olympus 3.1 mm BF-XP290 or an Olympus 4.2 mm BF-P290 bronchoscope. The entire process was recorded on video and stored as an AVI file. Two physicians who were blinded to the clinical history reviewed the laboratory data and bronchoscopy videos and scored them [11]. Nine sites were scored: the trachea, right main trunk, right upper lung lobe, right intermediate bronchial trunk, right middle lobe, right lower lobe, left main bronchus, left upper lobe (including the lingual lobe) and left lower lobe. Each site was scored according to four bronchoscopy visual features, two related to secretions – quantity and colour – and four related to airway mucosal appearance – oedema, folds or elevations, erythema and pallor. The colour of the discharge was scored according to the BronkoTest® sputum colour chart (with a range of 0–8). Mucosal oedema, folds or elevations, erythema and pallor were characterised by a severity scale of 0–2 (0 = none, 1 = mild, and 2 = moderate to severe). For the mucosal appearance of each site, a composite score of 0–3 was based on the number of sites affected (0 = nil present; 1 = less than half of the nine sites with a score of 1; 2 = more than half of the nine sites with a score of 1 or less than half of the nine sites with a score of 2; and 3 = more than half of the nine sites with a score of 2). In this study, clinicians carried out the scoring, and the calibration was performed by several experienced paediatric bronchoscopists.

### Statistical analysis

The statistical analysis was performed using GraphPad Prism 8.0 (GraphPad Software Inc., San Diego, CA, USA). Data for continuous variables were presented as mean ± standard deviation (SD), and if bias was present, the median was used. The unpaired t-test or nonparametric Mann–Whitney test was used for the two-group analysis of continuous variables. Categorical variables were assessed using Fisher’s exact test. Endoscopic bronchitis scores under different ventilation modes were evaluated using ROC curves. Survival was estimated using Kaplan–Meier analysis, and the log-rank test was used to compare the results. *P* < 0.05 was considered statistically significant.

## Results

### Baseline characteristics of the study population

Most of the patients (122, 53.28%) were male and aged = 12 months (142, 62.01%). Acute hypoxic respiratory failure was the most common cause of ICU admission (178 cases, 77.73%), followed by neurological diseases (20 cases, 8.73%) and trauma (19 cases, 8.3%). The pattern of infiltration seen on chest imaging (chest X-ray or CT) was classified as focal (infiltration limited to one lobe) or diffuse (involving both lungs or multiple lobes of one lung). Diffuse changes were found in 165 patients (72.05%). The detection rate of pathogens was 78.6%, and 345 pathogens were detected, including 181 bacterial strains (52.46%), 66 mycoplasma strains (19.13%), 88 virus strains (25.51%) and 10 fungal strains (2.90%). A total of 67 patients (29.26%) were mechanically ventilated, and 58 patients were on the machine for more than 48 h. The median APACHE II score was 11 (4–42), and the median SOFA score was 8 (5–12). The median length of hospital stay for all patients was 16 days, the median length of stay in the ICU was 10 days, and the number of days prior to admission (medical history) was 4 days. The total score under the endoscope was 9.66 ± 2.76; 60 patients (26.20%) underwent the lavage treatment within 24 h; and 60 patients (26.20%) underwent the treatment more than twice. Additionally, 128 patients (55.90%) had multiple organ involvement, and 22 patients (9.61%) died (Table 1).

**Table 1:** Baseline characteristics of patients (*n* = 229 )

Feature	
Age, n ( median )	
≦12 months	1 42 (3)
12–36 months	3 1 (2 0 )
3–6 years old	23 ( 3.83 )
Over 6 years old	33 ( 9 )
Gender, n (%)	
Male	122 (53.28)
Female	107 (46.72)
Reason for ICU admission, n (%)	
Respiratory failure	17 8 (7 7.73 )
Septic shock	3 ( 1.31 )
Nervous system disease	20 ( 8.73 )
Multiple trauma	19 ( 8.30 )
Genetic metabolism and congenital malformations	4 ( 1.75 )
Renal failure	2 ( 0.87 )
Other illnesses	3 ( 1.31 )
Disease pattern on chest radiography, n (%)	
Involvement of multiple lobes	1 65 (72.05)
Single lobe involvement	64 (27.95)
Ventilatory support, n (%)	
Noninvasive positive pressure ventilation	162 (70.74)
Mechanical ventilation	67 (29.26)
Intubation time	
0–48 h	9
> 48 h	58
Disease severity, median (IQR)	
APACHE II	11 (4–42)
S ofa	8 (5–12)
Days in hospital, d	1 6 ( 3-120 )
ICU days, d	1 0 ( 2–68 )
Days before admission	4 ( 0.042-90 )
Pathogen positive (n, %)	1 80 ( 78.6 )
Total number of detected pathogens, n	345
Bacteria (n, %)	181 ( 52.46 )
Mycoplasma (n, %)	6 6 (19.13 )
Virus (n, %)	88 (25.51)
Fungi (n, %)	10 (2.90)
Endoscopy and lavage ( ‾x ± s)	
Secretion quantity	4.52 ± 0.95
Secretion color	2.43 ± 0.90
Mucosal edema	1.47 ± 0.59
Mucosal folds or elevations	1.73 ± 0.94
Mucosal erythema	1.26 ± 0.55
Mucosal pale	1.19 ± 0.42
Endoscopy total score	9.66 ± 2.76
Number of BALs	4.52 ± 0.95
Do lavage within 24 h, n (%)	6 0 (2 6.20 )
> 2 lavages, n (%)	6 0 (2 6.20 )
Prognosis (n ,% )	
Complications of pneumonia	1 28(55.90)
Die	22(9.61)
Complications of BAL(n,%)	
Adverse event	27 (7.71%)
Transient breathing deterioration	5 (1.43%)
Difficulty sedation	5 (1.43%)
Minor bleeding	8 (2.29%)
Interrupt operation	2 (0.57%)
Serious adverse event	7 (2.0%)
Pneumothorax	3 (0.86%)
Tracheal intubation	4 (1.14%)
Severe bleeding	0 (0.0%)

### Analysis of characteristics of different ventilation cases

In the analysis of the characteristics of different ventilation methods (Table 2), the prevalence of male patients with invasive ventilation was significantly higher than that of patients with non-invasive ventilation (*P* < 0.0001). The APACHE II and SOFA scores of patients with invasive ventilation were higher than those of patients with non-invasive ventilation (*P* = 0.0468, *P* = 0.0006). Bronchoscopy within 72 h after ICU admission was lower in the invasive ventilation group than in the non-invasive ventilation group (*P* = 0.0492). In terms of ICU hospitalisation days, total hospitalisation days and ICU mortality, the scores in the invasive ventilation group were higher than those in the non-invasive group (*P* = 0.0004, *P* < 0.0001, *P* = 0.0024, respectively). The APACHE II and SOFA scores after FOB were lower than those before treatment (*P* < 0.0001). Comparison of early and late bronchoscopy and alveolar lavage treatment in terms of ICU hospitalisation days and total hospitalisation days found that the early FOB group had shorter stays than the late FOB group, and the differences were statistically significant (*P* < 0.0001, *P* = 0.0007) (Table 3).

**Table 2:** Analysis of the characteristics of cases with different ventilation methods

Patient characteristics	Invasive (*n* = 67)	Non-invasive (*n* = 162)	*P* value
Male, n (%)	53(79.10)	69 (42.59)	< 0.0001
≦ 1 y	39(58.21)	95(58.64)	0.9518
APACHE II at admission ( ‾x ± s)	12.40 ± 5.84	11.03 ± 4.18	0.0468
SOFA day 1 ( ‾x ± s)	7.55 ± 1.90	6.76 ± 1.40	0.0006
Number of diseased lobes, ( ‾x ± s)	2.81 ± 1.61	2.73 ± 1.49	0.7471
FOB within 24h after admission to ICU, n(%)	15 (21.31)	45 (29.63)	0.3988
FOB within 48 h after admission to ICU, n(%)	20 (26.23)	68 (43.83)	0.0862
FOB within 72 h after admission to ICU, n(%)	26 (36.07)	86 (53.70)	0.0492
ICU length of stay, ( ‾x ± s)	16.46 ± 11.00	11.66 ± 8.33	0.0004
Total days in hospital, ( ‾x ± s)	30.03 ± 21.57	18.77 ± 14.69	< 0.0001
ICU mortality, n(%)	13 (19.40)	9 (5.56)	0.0024

**Table 3:** Early and late FOB characteristics

Patient characteristics	24 h(*n* = 60)	> 24 h(*n* = 169)	*P* value
Male, n (%)	35(58.33)	87(51.67)	0.3712
≦1 y	31(51.48)	112(66.27)	0.0620
APACHE II variation ( ‾x ± s)	11.28 ± 5.81	11.28 ± 4.23	0.6261
SOFA change ( ‾x ± s)	7.23 ± 1.84	6.85 ± 1.55	0.4043
ICU length of stay, ( ‾x ± s)	9 ± 6.18	14.57 ± 9.97	< 0.0001
Total days in hospital, ( ‾x ± s)	18.40 ± 16.37	23.46 ± 18.02	0.0007
Mortality, n(%)	6(10)	16(9.47)	> 0.9999
Invasive ventilation days	5.47 ± 5.97	7.22 ± 8.40	0.4332

### Pathogenic analysis of different age groups and different ventilation modes

The pathogen ranking in all age groups was as follows. There were 66 cases (36.67%) of *Mycoplasma pneumoniae* (Mp), 47 cases (26.11%) of *Staphylococcus aureus*, 42 cases (23.33%) of *Haemophilus pneumoniae*, and 30 cases (16.67%) of *Streptococci*. In the infant group, 6 cases of *Catamora*, 7 cases of *Enterobacter* and 1 case of *Serratia* marcescens were detected. The viruses detected were respiratory syncytial virus (RSV) in 49 cases (27.22%), influenza B virus in 31 cases (17.22%) and influenza A virus in 8 cases (4.44%). The fungus detected was *Candida albicans* in ten cases (5.56%). *Stenotrophomonas maltophilia*, *Klebsiella pneumoniae, Acinetobacter baumannii* and *Pseudomonas aeruginosa* were detected in all age groups and were more common in patients less than 12 months old; however, the difference was not statistically significant. There were more patients aged 13–36 months with mycoplasma as the pathogen in the invasive group than in the non-invasive group, and the difference was significant (*P* = 0.0134); the comparison of the detection rate of mycoplasma in each age group showed a statistically significant difference, and the 13–36 months group had the highest detection rate (*P* < 0.0001); the total difference in the detection rate of pathogens in each age group was statistically significant (*P* = 0.0428). There was one case of *Bordetella pertussis* in the group less than 1 year old and one case of actinomycetes in the group more than 6 years old. Additionally, there was one case of *Bordetella pertussis* in the group less than 1 year old and one case of actinomycetes in the group more than 6 years old. Mycoplasma: (13–36 months) invasive and non-invasive comparison (*P* = 0.0134); comparison of detection rates in each age group (*P* < 0.0001). There was a statistically significant difference in the total detection rate of pathogens in each age group (*P* = 0.0428) (Table 4).

**Table 4:** Microorganisms detected in samples obtained by bronchoscopy at different ages

Type of microorganism	12 months		13–36 months		4–6 years old		over 6 years old	
	Invasive *n* = 38	Non-invasive *n* = 103	Invasive *n* = 11	Noninvasive *n* = 20	Invasive *n* = 8	Non-invasive *n* = 15	Invasive *n* = 10	Noninvasive *n* = 24
Bacteria								
Staphylococcus aureus	4 (1 0.53 )	22 (2 1.36 )	1 ( 9.09 )	2 (1 0.0 )	1 (1 2.50 )	5 (3 3.33 )	5 (5 0. 0 )	7 (29.17)
Streptococcus pneumoniae	5 (13.16)	15 (14.56)	0 (0)	3 (15)	0 (0)	2 (13.33)	2 (20.0)	3 (12.5)
Mycoplasma pneumoniae	8 (21.05)	20 (19.42)	7 (63.64)	3 (15)	2 (25)	5 (33.33)	4 (40)	17 (70.83)
Haemophilus	8 (21.05)	20 (19.42)	0 (0)	3 (15)	0 (0)	5 (33.33)	1 (10)	5 (20.83)
Stenotrophomonas maltophilia	2 (5.26)	3 (2.91)	1 (9.09)	1 (5)	0 (0)	0 (0)	0 (0)	1 (4.17)
Klebsiella pneumoniae	3 (7.89)	9 (8.74)	1 (9.09)	0 (0)	2 (25)	1 (6.67)	0 (0)	3 (12.5)
Acinetobacter baumannii	1 (2.63)	6 (5.83)	1 (9.09)	1 (5)	1 (12.5)	1 (6.67)	1 (10)	3 (12.5)
Pseudomonas aeruginosa	1 (2.63)	2 (1.94)	2 (18.18)	0 (0)				
Catamora	3 (7.89)	3 (2.91)						
Streptococcus vestibules	0 (0)	1 (0.97)						
Enterobacter	2 (5.26)	5 (4.85)						
Serratia marcescens	0 (0)	1 (0.97)						
Virus								
Influenza A	0 (0)	0 (0)	4 (36.36)	0 (0)	0 (0)	1 (6.67)	0 (0)	3 (12.5)
Influenza B	0 (0)	22 (1.94)		0 (0)	0 (0)	2 (13.33)	3 (30)	4 (16.67)
respiratory syncytial virus	14 (36.84)	30 (29.13)	1 (9.09)	3 (15)	0 (0)	1 (6.67)		
fungi								
Candida albicans	3 (7.89)	1 (0.97)	0 (0)	0 (0)	0 (0)	2 (13.33)	0 (0)	4 (16.68)
total positive	32 (84.21)	79 (76.70)	9 (81.82)	12 (60)	5 (62.5)	11 (73.33)	8 (80)	24 (100)

### Analysis of the relationship between microscopic score and severity of disease

The endoscopic mucosal oedema, erythema and pallor scores and the total score of the invasive ventilation group were higher than those of the non-invasive ventilation group (*P* = 0.0052, *P* = 0.0018, *P* = 0.0113, and *P* = 0.0031), consistent with the SOFA score (*P* = 0.0006). In the group where FOB and BAL were performed within 1 day of ICU admission, the secretion colour score was lower than that of the late BAL group.

(*P* = 0.0088) (Table 5).

**Table 5:** The relationship between the microscopic score and the severity of the disease

	total	invasive	non-invasive	*P*	within 1 day	1 day later	*P*
Secretion quantity	4.52 ± 0.95	4.49 ± 0.96	4.52 ± 0.95	0.8161	4.37 ± 1.09	4.57 ± 0.89	0.7201
Secretion color	2.43 ± 0.90	2.37 ± 0.98	2.45 ± 0.87	0.5560	2.17 ± 0.94	2.52 ± 0.87	0.0088
Mucosal edema	1.47 ± 0.59	1.58 ± 0.67	1.43 ± 0.54	0.0052	1.49 ± 0.61	1.45 ± 0.55	0.6897
Mucosal folds or elevations	1.73 ± 0.94	2.13 ± 0.83	1.62 ± 0.87	0.2014	1.50 ± 0.84	1.93 ± 0.88	0.3163
Mucosal erythema	1.26 ± 0.55	1.33 ± 0.59	1.25 ± 0.52	0.0018	1.21 ± 0.48	1.30 ± 0.55	0.3999
Mucosal pale	1.19 ± 0.42	1.22 ± 0.42	1.17 ± 0.42	0.0113	1.07 ± 0.25	1.24 ± 0.46	0.0604
total score	9.66 ± 2.76	9.09 ± 4.14	7.28 ± 4.16	0.0031	9.22 ± 2.77	9.82 ± 2.74	0.1479
APCHE II	11.43 ± 4.76	12.36 ± 5.86	11.05 ± 4.18	0.0580	11.70 ± 6.07	11.34 ± 4.21	0.6129
SOFA	6.99 ± 1.60	7.55 ± 1.90	6.76 ± 1.40	0.0006	7.28 ± 1.87	6.89 ± 1.49	0.1006

### Area under receiver operating characteristic curve of endoscopic changes and survival curve under different ventilation modes

**Fig. 2:**
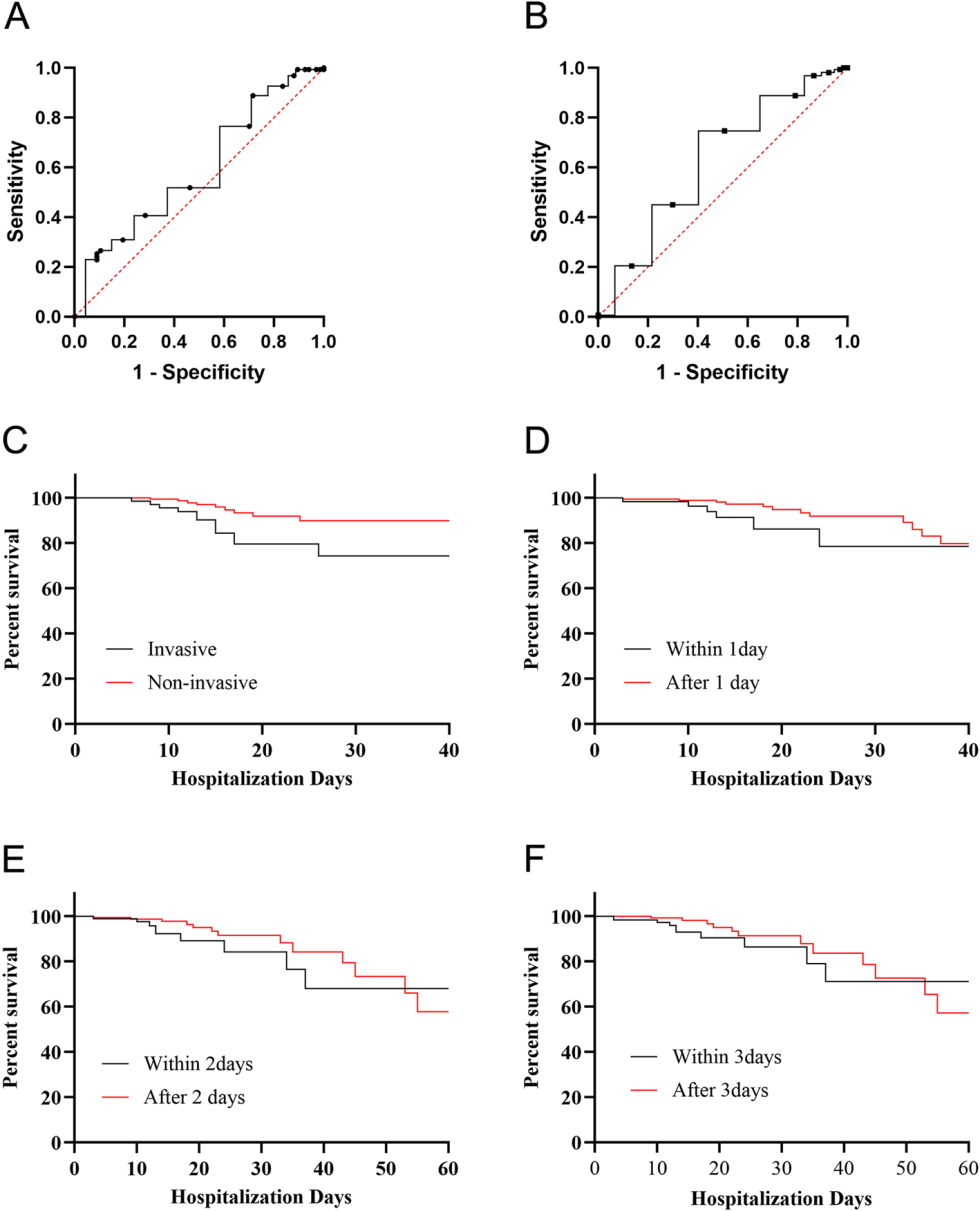
ROC curve of endoscopic changes and Survival curve under different ventilation modes Note:A:Total score and invasive ROC curve, aROC was 0.5897, 95%CI (0.5093-0.6700), *P* = 0.0328; B:The ROC curve of score and sofa, aROC was 0.6265, 95%CI (0.5446–0.7083), *P* = 0.0026. C: Survival curve of fiberoptic bronchoscopy within 1 day and 1 day after admission, Log-rank ( Mantel-Cox ) test, *P* = 0.3108.D: invasive and non-invasive ventilation survival curve, Log-rank ( Mantel-Cox ) test *P* = 0.0061.E: The survival curve of fiberoptic bronchoscopy within 2 days and 2 days after admission, Log-rank ( Mantel-Cox ) test, *P* = 0.3038. F: Survival curve of fiberoptic bronchoscopy within 3 days and 3 days after admission, Log-rank ( Mantel-Cox ) test *P* = 0.3974

In the study of the role of endoscopy scores under different ventilation modes, mucosal erythema, pallor, oedema and the total score obtained higher area under receiver operating characteristic (aROC) curves and were statistically significant (aROC = 0.7715, 95% confidence interval (CI) (0.6554–0.8877), *P* = 0.002; aROC = 0.6000, 95% CI (0.5166–0.6833), *P* = 0.0199; aROC = 0.5911, 95% CI (0.5091–0.6732), *P* = 0.0329; and aROC = 0.5897, 95% CI (0.5093–0.6700), *P* = 0.0328, respectively); for the SOFA score, the predictive value of aROC was also higher (aROC = 0.6265, 95% CI (0.5446–0.7083), *P* = 0.0026) (Table 6; Fig. 2A, B).

**Table 6:** Predicted ROC for endoscopic changes with and without ventilator bronchoscopy

Model	aROC	Std.Error	95% confidence interval	*P* value
Secretion quantity	0.5266	0.04333	0.4417–0.6115	0.527
Secretion color	0.5308	0.04431	0.4439–0.6176	0.464
Mucosal edema	0.5911	0.04186	0.5091–0.6732	0.0329
Mucosal folds or elevations	0.6683	0.1229	0.4273–0.9092	0.205
Mucosal erythema	0.7715	0.05925	0.6554–0.8877	0.002
Mucosal pale	0.6	0.04253	0.5166–0.6833	0.0199
total score	0.5897	0.041	0.5093-0.6700	0.0328
APCHE II	0.557	0.04269	0.4733–0.6407	0.1751
SOFA	0.6265	0.04176	0.5446–0.7083	0.0026

*aROC *Area under the curve

The timing of FOB examination and the survival curve analysis of different ventilation methods are shown in Fig. 2. The Kaplan–Meier analysis and log-rank test showed that the survival rate of the non-invasive ventilation group was higher than that of the invasive ventilation group (*P* = 0.0061). There was no significant difference in the survival rate among different timings of FOB examination (*P* > 0.05) (Fig. 2C–F).

### Complications

A satisfactory sedative effect lasting until the end of the bronchoscopy was achieved for most patients after one dose of a combination of sedative drugs. Complications can include a brief deterioration in breathing, slight bleeding and pneumothorax. Invasive mechanical ventilation was required in four cases after the bronchoscopy. Pneumothorax occurred in three patients who underwent invasive alveolar lavage, and laryngotracheal spasm occurred in five patients; these symptoms were relieved by sedation and oxygen inhalation. There were no operation-related deaths. Four patients died during hospitalisation, including two cases of severe *Staphylococcus aureus* pneumonia and two cases of acute respiratory distress syndrome (ARDS, Table 1).

## Discussion

The overall beneficial effect of microbiological diagnostic bronchoscopy on clinical outcomes in pneumonia is well known. In previous studies of patients without human immunodeficiency virus (the same as the current study’s population), the aetiological diagnosis of FOB and BAL was 52–84% [12]. The present study found that the pathogen detection rate reached 78.6%, which is higher than that found by Panse et al. (67.2%) [13] and Ma et al. (75%) [14]. This was probably because of the development of diagnostic techniques such as pathogenic metagenomic technology in recent years [15]. However, these techniques have limitations in clinical application due to various factors, such as cost [16].

In this study, the pathogen with the highest detection rate was mycoplasma, and it was the highest in each age group. The 13–36 month group had the highest incidence, and the difference was statistically significant. The main pathogens detected were Mp, RSV, *Staphylococcus aureus*, *Haemophilus influenza (Hi) B virus*, and *Streptococcus pneumoniae* (Sp). Ma et al. [14] found that 53.7% of the respiratory pathogens detected in patients were viruses, 32.9% were bacteria, and 13.4% were Mp; the reason for this difference in the results of this study and that of Ma et al. may be related to the different sample sizes. The sample size of this study is relatively larger than that of Ma et al.’s study. Slightly different from the findings of the study conducted by Ma et al., the top five pathogens in this study were RSV, Sp, Hi, Mp, and adenovirus (AdV). It is worth noting that in this study, RSV was a common virus in critically ill patients under 1 year old, followed by the influenza virus. No AdV was detected, however, and the fungal population was surprisingly low. Conversely, this virus was found to be more common in the research conducted by Ma et al. and Huang et al. [14, 17, 18], especially with the development of pathogenic metagenomic technology. The reason for this difference may be related to disease severity and regional factors in the included patients.

There is still no well-recognised endoscopic scoring tool for assessing the severity of pneumonia in children. The most commonly used method is to predict the severity of pulmonary infection by clinical indicators rather than intrapulmonary manifestations. Zhao et al. [19] constructed a nomogram to predict the development of plastic bronchitis in children with refractory mycoplasma pneumoniae pneumonia (RMPP) by studying six variables: peak body temperature, neutrophil ratio, PLT, IL-6, LDH and atelectasis. In Cheng’s study [20], indicators such as lactate dehydrogenase, albumin, neutrophil ratio and high fever were used. None of these indicators can objectively reflect the degree of inflammatory changes in the lung, and most of the previous studies were single-centre studies [21].

As early as 1989, Thompson developed a semi-quantitative bronchoscopy scoring system based on mucosal fragility, oedema and erythema to describe secretions of intraluminal airway inflammation [22]; however, it is not widely used. It was not until 2018 that researchers, such as Thomas, developed a bronchial inflammation scoring tool [11]. In 2020, a team led by Thompson validated six visual characteristics – secretions (number and colour) and mucosal appearance (erythema, pallor, elevation and oedema) – in a prospective study and concluded that secretion volume and colour, and mucosal oedema and erythema were significantly associated with BAL neutrophils [23]. Lung inflammation can be reflected by the endobronchial lining and secretions. However, a large number of studies failed to verify this, and there are few reports on the severity of pneumonia and the results of altered bronchoscopy.

In studies conducted by other researchers, BALF microbial cell counts increased with increasing bronchoscopy grades of secretions and were associated with bronchial inflammation [11]. Microbial growth in BALF was greater in patients with bronchoscopy grade IV secretions and above [24]. Nursoy et al. found that the incidence of culture-detected microorganisms in BALF of patients with bronchoscopy grade IV secretions and above increased. With an increasing bronchoscopy secretion score, the first second of expiratory volume and forced vital capacity values gradually decreased, and the bronchial Bhalla score and bronchial mucosal inflammation increased [25]. However, no study has linked scoring tools to disease severity. In this study, the endoscopic mucosal oedema, erythema and pallor scores and the total score in the invasive ventilation group were higher than those in the non-invasive ventilation group. In the FOB and BAL groups, within 1 day of admission to the ICU, the colour score of the secretions was lower than that in the operation group after 1 day. When investigating the effect of endoscopic scores under different ventilation modes, mucosal erythema, pallor, oedema and the total score had better aROCs, which was basically consistent with the performance of the SOFA score aROCs. However, most of the aROCs in this study were no higher than 0.8, and the predictive value was not perfect, which may be related to the small sample size, therefore further prospective studies are still needed. Moreover, as this study is a retrospective study, the percentage of neutrophils in BAL could not be collected, so the validity of this tool needs to be further verified.

Bronchoscopy procedures are often avoided in critically ill patients, especially those on mechanical ventilation because of the risk of complications. However, it has also been reported that bronchoscopy can reduce the incidence of pneumonia, length of hospital stay and mortality in patients who have undergone tracheostomy [26]. Other studies have shown that bronchoscopy for aspiration injury pneumonia can help to reduce ICU length of stay, hospital costs and even mortality by addressing airway problems [27]. The present study also showed that patients in the invasive ventilation group had higher APACHE II scores, SOFA scores, lengths of ICU stay, total lengths of stay and ICU mortality than patients in the non-invasive ventilation group, and early FOB could reduce the length of ICU stay and total length of stay; the lavage treatment was completed within 24 h in 60 cases (26.20%), multiple organs were involved in 128 patients (55.90%), and 22 patients (9.61%) died.

Studies have shown that bronchoscopy within 24 h is beneficial for clinical outcomes in mechanically ventilated patients with aspiration pneumonia [28]. These studies suggest that therapeutic bronchoscopy may benefit patients with severe pneumonia by reducing airway inflammation. The results of the current study showed that early FOB can shorten the length of ICU stay; however, there was no difference in mortality. The survival analysis indicated that the survival rate of the early FOB group was lower than that of the late FOB group. This indicates that bronchoscopy may not improve the survival rate of ICU patients with severe pneumonia. A retrospective analysis of the clinical data of the death cases revealed that they were not related to bronchoscopy. Two cases underwent ECMO treatment for severe ARDS, one had intracranial haemorrhage, and four had multiple malformations, which may be related to the severity of the disease and insufficient sample size. Related studies have also speculated that necrotizing bronchitis and diffuse alveolar damage of bronchioles may occur early in critically ill patients [13]. These factors contribute to poor tolerance of BAL and increase the risk of complications. A recent study of AdV pneumonia also showed that there was no statistically significant difference in the duration of fever and hospital stay between patients in the severe group who received BAL in the early stages and those who received BAL in the later stages [18]. It is even speculated that receiving BAL in the early stages may increase the chance of mechanical ventilation and the occurrence of surgical complications in severe cases. As this study shows, adverse events during or after BAL in ICU patients are not uncommon. According to this study, the occurrence of serious complications, such as pneumothorax, may be related to the inexperience of the performing physician and the longer operation time. Studies have concluded that the inexperience of physicians performing BAL may be a major predictor of clinically significant adverse events in non-intubated patients [8].

## Conclusion

Fibreoptic bronchoscopy combined with BAL appears to be an effective treatment for severe pneumonia and also increases its detection rate. In addition, early BAL can reduce hospitalisation and time spent in ICU; however, it cannot reduce mortality, which may be related to the severity of the disease. This study also demonstrated that the endoscopic score can reflect the severity of lung inflammation. However, the sample in the current study was small, so the authors will conduct a multi-centre study with a larger sample in the future.

## Data Availability

All data generated or analyzed during this study are included in this published article.

## Abbreviations

FOB: Fiberoptic bronchoscopy
BAL: Bronchoalveolar lavage
BALF: Bronchoalveolar lavage fluid
PICU: Pediatric intensive care unit
APACHE: Acute Physiology and Chronic Health Assessment
SOFA: Sequence Overall organ failure assessment
